# Exploring the Potential of Adjuvant CDK4/6 Inhibitors in Hormone Receptor-Positive Early Breast Cancer: A Consistent Approach for All

**DOI:** 10.3390/cancers17040561

**Published:** 2025-02-07

**Authors:** Jianbin Li

**Affiliations:** 1 Senior Department of Oncology, Fifth Medical Center of PLA General Hospital, Beijing 100071, China; docli1018@sina.com; 2 Department of Medical Molecular Biology, Beijing Institute of Biotechnology, Academy of Military Medical Sciences, Beijing 100071, China

**Keywords:** early breast cancer (EBC), hormone receptor-positive/human epidermal growth factor receptor 2-negative (HR+/HER2-), CDK4/6 inhibitors, NATALEE, monarchE

## Abstract

Background/Objectives: Hormone receptor-positive, HER2-negative breast cancer is the most common subtype, with endocrine therapy as the standard treatment. Despite the advancements in adjuvant endocrine therapy, recurrence remains a challenge, particularly in high-risk patients. Recent trials on cyclin D kinase 4/6 (CDK4/6) inhibitors in adjuvant therapy have shown promise in reducing early recurrence and improving survival. Methods: This review analyzes the clinical evidence supporting the use of CDK4/6 inhibitors, focusing on the NATALEE and monarchE trials, which demonstrate comparable efficacy and manageable safety profiles for ribociclib and abemaciclib. Results and Conclusions: Ribociclib, with its broader applicability and impact on the decision making for axillary lymph node surgery, may be the preferred option in high-risk populations. The review also addresses unanswered clinical questions and highlights the need for ongoing research to optimize the adjuvant therapy strategies.

## Simple Summary

Cyclin D kinase 4/6 inhibitor has changed the pattern of adjuvant endocrine therapy for early breast cancer. While the results of the NATALEE and monarchE studies are not completely consistent. It is necessary to sort out the differences in these two clinical studies and help to address these unsolved clinical questions

## 1. Introduction

Hormone receptor (HR)-positive human epidermal growth factor receptor 2 (HER2)-negative breast cancer is the most common molecular subtype of breast cancer [1], accounting for about 70% to 75% of cases [2]. The majority of breast cancers (≈80%) are non-metastatic early breast cancers, and the treatment of early breast cancers is aimed at a cure. HR-positive, HER2-negative early breast cancers are treated with surgery with or without radiotherapy or chemotherapy, followed by adjuvant endocrine therapy for 5 to 10 years [3]. However local and/or distant recurrence remains a significant problem in this patient population, and the prevention of early and late recurrence is an equally important consideration when determining the adjuvant treatment options for HR+/HER2-EBC patients [4,5]. The risk of recurrence is highest in the first 5 years after diagnosis, and depending on the risk of recurrence and the likelihood of benefit from chemotherapy, treatment with adjuvant chemotherapy may be recommended. However, 50% of patients with ER+EBC recurrence will recur at a late stage (5 years after diagnosis). After completion of 5 years of standard endocrine (ET) therapy, recurrence occurs in 27% to 37% of patients with stage II and 46% to 57% of patients with stage III and may recur up to 20 years after diagnosis [6].

CDK4/6 inhibitors are cell cycle protein-dependent kinase inhibitors that block the phosphorylation of Rb proteins, thereby inhibiting the cell transition from the G1 to S phase and achieving the inhibition of tumour cell proliferation [7]. Trials of ribociclib, palbociclib, abemaciclib, and dalpiciclib in advanced breast cancer have demonstrated the ability to improve progression-free survival in patients with HR+/HER2- advanced breast cancer [8,9,10,11,12,13,14]. But only ribociclib and abemaciclib were shown to have a significant overall survival benefit [15,16,17,18], and it is worth noting that ribociclib achieved a significant overall survival benefit in all three phase III trials. However, the effect of adjuvant therapy with CDK4/6 inhibitors has been inconsistent in early-stage breast cancer. In the PALLAS trial [19] and the PENELOPE-B trial [20], palbociclib in combination with endocrine therapy did not show a significant aggressive disease-free survival benefit. In contrast, significantly improved invasive disease-free survival was demonstrated in the monarchE trial [21] of 2 years of abemaciclib adjuvant therapy and the NATALEE trial [22] of 3 years of ribociclib-adjuvant therapy. Currently, there are two CDK4/6 inhibitors that have been approved globally for HR+/HER2- early breast cancer. These are abemaciclib (approved in October 2021) and ribociclib (approved in September 2024). However, the indications for which these two drugs have been approved vary. This review examines the characteristics of the risk of disease recurrence in early-stage breast cancer, the evolution of the landscape of adjuvant endocrine therapies, the CDK4/6 inhibitor intensive adjuvant therapy population, a comprehensive comparison of the NATALEE and monarchE trials, the development of the future therapeutic landscape, the biomarkers and mechanisms of action, and a detailed account of the use of CDK4/6 inhibitors in early breast cancer.

### 1.1. HR+/HER2- Early Breast Cancer Disease Recurrence Risk Profile

In women diagnosed with early-stage breast cancer, many patients can achieve disease-free survival with surgical excision and radiotherapy, but the risk of recurrence is a significant lifelong problem and is relatively common. Local recurrence occurs in 8% to 10%, and distant metastases in 15% to 30% [23]. The recurrence of breast cancer may occur early (usually defined as within 5 years of diagnosis) or late (more than 5 years). The risk of recurrence in patients with hormone receptor-positive (HR+) tumours lasts longer than in patients with HR- tumours [24] and is reflected in the differences in survival [25]. According to a meta-analysis of nearly 150,000 women in 200 randomised clinical trials by the EBC Trialists’ Collaborative Group (EBCTCG), approximately 36% and 20% of patients with early-stage breast cancer who do not receive any adjuvant systemic therapy will recur and die from their breast cancer, respectively, during the 5-year follow-up period. In addition, recurrence and breast cancer-related death in patients with hormone receptor (HR)-positive EBC continued to occur 5 years after surgery, with only 45% of patients reporting no recurrence at 15 years of follow-up [26].

Adjuvant therapy, including cytotoxic agents and endocrine therapy, reduces local and distant recurrence and improves the overall survival (OS) in patients with early-stage breast cancer. The selection of adjuvant therapy needs to be based on the individual risk of recurrence. Accurate assessment of the risk of recurrence in these patients is critical, as inaccurate estimates of prediction may lead to over- or undertreatment, either of which can result in adverse outcomes for patients [27]. The IRIDE (hIGh Risk DEfinition in breast cancer) Working Group summarises the current characteristics associated with predicting the recurrence of hormone-positive/HER2-negative early breast cancer [28]. The assessment of the individual recurrence risk needs to be guided by a combination of multifactorial clinical, pathological and genomic predictive and prognostic factors for both the patient and the tumour, such as tumour stage, histopathological grading, proliferative indices such as Ki67, age, menopausal status, comorbidities, and multigene test recurrence scores. Since no single test alone can provide an accurate prognosis, it is recommended that multiple prognostic tools and factors be used for early breast cancer to avoid overtreatment or inappropriate treatment. The following paragraphs describe the major classical clinicopathological and molecular prognostic factors for breast cancer.

### 1.2. Clinical and Tumour Pathology Prognostic Factors in Routine Patients

Younger age (usually defined as less than 35–40 years) is associated with a poor prognosis in breast cancer [29]. Patients with comorbidities have poorer survival, which tends to occur in older patients [30].

Tumour size (T), lymph node involvement (N), and distant metastasis (M) are the factors that have the greatest impact on prognoses. These three features constitute the TNM staging system [31]. There is a clear correlation between an increase in tumour size and the number of lymph nodes involved and the risk of metastasis [32]. The tumour grade (the Nottingham grading system) is one of the most commonly used grading systems for breast cancer, and a study with a 25-year follow-up showed that an increase in the tumour grade was significantly associated with the risk of recurrence, with the correlation being more pronounced in the first 5 years [33]. Ki-67 is an immunohistochemical marker of cell proliferation, and generally speaking, the higher the level of Ki-67 expression, the worse the prognosis of breast cancer. There is no consensus on the threshold of Ki-67 that defines high risk [34]. In the inclusion criteria of the NATALEE and monarchE trials, Ki-67 ≥ 20% was chosen as the threshold for high risk under certain conditions.

### 1.3. Tools for Assessing the Risk of Recurrence After Breast Cancer Surgery Based on the Clinicopathological Features

Several breast cancer prognostic scoring systems have been developed, including the Nottingham Prognostic Index (NPI), immunohistochemical 4 (IHC4), Adjuvant! Online (AOL), clinical treatment score post 5 years (CTS5), and so on. However, these postoperative recurrence risk assessment tools were developed at the end of the last century and the beginning of this century, mostly to assess the effect of classical endocrine therapy, and did not take into account the impact of CDK4/6 inhibitors after their application in the adjuvant phase.

The Nottingham prognostic index (NPI) uses lymph node stage, tumour size, and the histological grade of early primary breast cancer as parameters to estimate patient survival and prognosis in patients receiving adjuvant therapy [35]. The immunohistochemical 4 (IHC4) score is based on the conventional IHC diagnostic and prognostic scores for ER, PR, HER2, and Ki-67, and a recurrence risk score is derived by assigning different weights to each of the IHC parameters to assess the risk of postoperative recurrence in patients with breast cancer [36]. Adjuvant! Online (AOL) is a clinicopathological system developed by the National Cancer Institute that was developed using the Surveillance, Epidemiology, and End Results (SEER) registry, and based on the patient’s baseline information (age, menopausal status), pathological information (tumour size, lymph node status, ER and PR status), and after other comorbidities. The system automatically gives the patient a 10-year risk of recurrence and the expected outcome of different treatments (adjuvant chemotherapy, endocrine therapy) [37]. The clinical treatment score post 5 years (CTS5), which includes four variables, namely age, tumour size, lymph node status, and tumour grade, estimates the risk of distant recurrence at 5 years, and helps to identify patients who could benefit from extended endocrine therapy (>5 years) (https://cts5-calculator.com/ (accessed on 12 December 2024)) [38]. In addition, PREDICT [39], CanAssist Breast [40], and iCanPredict [41] have been reported by various regions based on the development of postoperative recurrence risk assessment tools.

### 1.4. Genomic Analysis of Prognosis in Early Breast Cancer

In recent years, in addition to the traditional clinicopathological factors, several genomic prognostic tests have been developed to estimate the risk of recurrence of breast cancer and to help decide on the decision to apply adjuvant chemotherapy. These include Oncotype DX RS (21 genes) [42], MammaPrint (70 genes) [43], EndoPredict [44], Prosigna/PAM50 [45], and the Breast Cancer Index (BCI) [46], and were tested in prospective clinical trials such as TAILORx [47], MINDACT [48], RxPonder [49], and OPTIMA [50]. There was no requirement for genomic analyses in the monarchE trial, and in the NATALEE trial, Oncotype DX RS, Prosigna/PAM50, MammaPrint, and EndoPredict high-risk populations were available for inclusion in patients with stage IIA N0 histological staging grade 2.

More recently, the integration of genomic and clinical characteristics is expected to guide the use of adjuvant chemotherapy more accurately, e.g., the RSClin model integrates the 21-gene recurrence score (RS) with the tumour grade, tumour size, and age [51], which improves the estimate of the risk of distant recurrence compared to the use of the 21-gene and clinicopathological factors alone [52], which provides a new line of enquiry for the more accurate assessment of the risk of recurrence in the future.

In summary, the prediction of recurrence risk is intended to guide the treatment choice [53], and as more and more treatment options become available, the prediction of recurrence risk and risk thresholds used to guide treatment decisions are evolving. The factors guiding adjuvant treatment selection at this stage have evolved from TNM staging to a combined assessment of patient risk via TNM staging combined with tumour pathology and genetic features, which is essential to avoid over- or undertreatment.

## 2. Evolution of Endocrine Therapy in HR+/HER2- Early Breast Cancer

The traditional endocrine therapy medications mainly include selective oestrogen receptor modulators (SERMs) such as tamoxifen (TAM) and toremifene as well as aromatase inhibitors (AIs) such as non-steroidal letrozole and anastrozole (NSAI), and steroidal exemestane. The benefits of adjuvant therapy with TAM and AIs have been demonstrated in large meta-analyses by the EBCTCG [26,54]. The benefits of intensive adjuvant endocrine therapy include the prolonged duration of adjuvant endocrine therapy [55], combination OFS [56], combination osteoprotegerics [57], and combination CDK4/6 inhibitor-targeted therapy [19,58,59]. In this paper, we focus on the data of intensive treatment with CDK4/6 inhibitors.

### 2.1. Mechanism of Action of CDK4/6 Inhibitors in Early Breast Cancer

**Figure 1:**
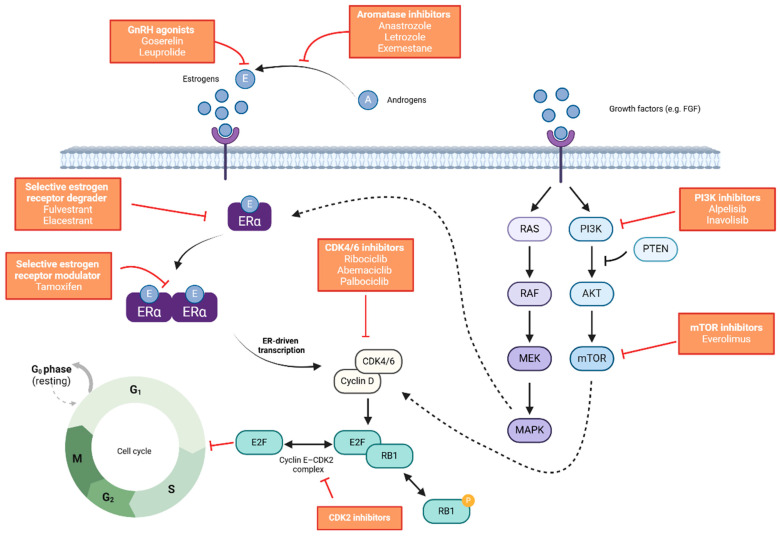
Mechanisms of action of endocrine and targeted therapies used for the treatment of ER+/HER2- metastatic breast cancer. Figure is created in BioRender. A, androgens; AKT, protein kinase B; CDK, cyclin-dependent kinase; E, estrogens; E2F, early region 2 binding factor; ER, estrogen receptor; FGF, fibroblast growth factor; GnRH gonadotropin-releasing hormone; MAPK, mitogen-activated protein kinase; MEK, mitogen-activated protein kinase kinase; mTOR, mammalian target of rapamycin; P, phosphate; PI3K, phosphatidylinositol 3-kinase; PTEN, phosphatase and tensin homolog; RAF, rapidly accelerated fibrosarcoma; RAS, rat sarcoma; RB1, retinoblastoma tumour suppressor protein.

Hormone-based treatments for oestrogen receptor-positive tumours have been demonstrated to deplete oestrogen production, interrupt oestrogen receptor signalling, degrade oestrogen receptors, or alter oestrogen receptor-regulated signalling or proliferation pathways (Figure 1). Breast cancer recurrence can be driven by a number of different mechanisms. Early recurrence may be fuelled by replicating circulating tumour cells, whereas late recurrence may be triggered by the awakening of dormant micrometastatic disease [60]. A longer duration of adjuvant endocrine therapy has been shown to have an impact on reducing late recurrence, including distant recurrence [61]. The extension of cell cycle arrest may be important, especially in the treatment of HR+ disease, where tumour cell dormancy can be long and 50% of recurrences occur after 5 years. The monarchE trial has only demonstrated a reduction in early recurrences, with efficacy sustained after two years of treatment. Longer follow-up analyses are still needed to assess whether abemaciclib also reduces the risk of late relapse. The NATALEE trial is designed to provide 3 years of ribociclib treatment to this broader patient population. This is the longest course of CDK4/6 inhibitor treatment for EBC and was chosen to maximise the exposure of circulating tumour cells to ribociclib. This was performed to move these cells from the cell cycle arrest phase into senescence (stable cell cycle arrest), even after treatment with ribociclib was discontinued. The prolongation of cell cycle arrest reduces the recurrence due to circulating tumour cells up to 20 years after diagnosis [53,62].

Ribociclib has a unique mechanism of action that has been shown to achieve the highest free drug concentrations and selectively target CDK4, thereby prolonging CDK4 target inhibition [63,64]. The results from preclinical studies suggest that CDK4 inhibition can contribute to senescence after growth arrest and prompt an immune response in cancer cell lines through on-target activity [65]. In an exploratory clinical analysis of immune responses using peripheral blood mononuclear cell samples from the RIBECCA trial, ribociclib had a significant effect on the peripheral innate and adaptive immune responses [66]. Gene set enrichment analysis during treatment with RIB revealed the upregulation of the signatures associated with an activated adaptive immune system and a decrease in immunosuppressive cytokine signalling. There was a significant increase in circulating CD4+ T cells (*p* = 0.006) and a decrease in peripheral immunosuppressive cell populations. Furthermore, in advanced breast cancer, ribociclib showed a carry-over effect, resulting in a long-term OS benefit [15,16,17], which may be due to ribociclib’s ability to alter tumour biology and induce immunomodulatory effects, thereby influencing the response to subsequent therapies. These immune effects can be considered indirect, as they primarily result from changes in tumour biology following CDK4/6 inhibition. Preclinical studies and exploratory clinical analyses, such as the RIBECCA trial, suggest that ribociclib may stimulate both innate and adaptive immune responses in the tumour microenvironment. This is likely to be mediated by an on-target activity that induces senescence in cancer cells, facilitating the recognition and clearance of these senescent cells by the immune system. In addition, a pooled analysis of the MONALEESA-2, -3, and -7 trials showed a trend towards the improved efficacy of subsequent therapies after first-line ribociclib plus endocrine therapy, suggesting a post-treatment effect of ribociclib [67]. These unique properties of ribociclib may have the potential to prevent both early and late recurrence, respectively, either through the direct elimination of replicating tumour cells or by locking dormant cells in a senescent state to facilitate immune-mediated clearance. Based on the results of these preclinical and clinical studies, we speculate that ribociclib may induce the senescence of micrometastatic tumour cells and induce an anti-tumour immune response that ultimately removes them. However, in terms of adverse events (AEs), neutropenia is a well-documented side effect of CDK4/6 inhibitors, including ribociclib. This is primarily due to the inhibition of CDK4, which interferes with normal haematopoietic cell division and leads to a reduction in neutrophil counts. Neutropenia is a direct effect of CDK4/6 inhibition on bone marrow function and is not caused by immune senescence mechanisms. While the ability of ribociclib to induce immune responses may contribute to its therapeutic effects, these immune changes are not directly responsible for neutropenia or other haematological AEs observed in clinical practice.

### 2.2. Inconsistent Results for Adjuvant CDK4/6 Inhibitor Therapy in HR+/HER2- Early Breast Cancer

Ribociclib, palbociclib, abemaciclib, and dalpiciclib in advanced breast cancer showed improved progression-free survival in patients with HR+/HER2- advanced breast cancer Christos Papadimitriou and overall survival [15,16,17,18]. In early-stage breast cancer, the three CDK4/6 inhibitors with currently published results have inconsistent effects (Table 1).

Two phase III studies of palbociclib in early breast cancer, PALLAS and PENELOPE-B, were conducted. The negative results of these two trials suggest that palbociclib is not suitable for use in early-stage breast cancer, but the exact reasons for the failures are unknown and further clinical studies are needed.

Abemaciclib is the first CDK4/6 inhibitor to achieve positive results in the adjuvant treatment of early breast cancer. With the positive results, abemaciclib was first approved by the FDA in October 2021 for the adjuvant treatment of early-stage breast cancer in a population limited to those with Ki-67 ≥ 20% in Cohort 1, which comprised 51% of Cohort 1 in the monarchE trial. Subsequent descriptive analyses at longer follow-ups showed the continued separation of the Kaplan–Meier curves for the two treatment groups, with a trend towards widening and 5 years of DFS: 83.6% vs. 79.4% (HR = 0.68, 95% CI 0.599–0.772, *p* < 0.001) [70]. This suggests that the carry-over effect remains after abemaciclib is discontinued, followed by the FDA’s August 2023 expansion of its indication to the overall Cohort 1 population. The overall survival data from the trial are currently immature and show a trend towards an OS benefit in the overall population.

The NATALEE trial of ribociclib also achieved positive results. In September 2024, ribociclib was approved by the FDA for the adjuvant treatment of early-stage breast cancer, and in a slightly different indication than abemaciclib, this approval was for the overall population of the NATALEE trial. The ESMO meeting in 2024 updated the results of the four-year follow-up of NATALEE, with a median follow-up time of 44.2 months; all the patients were no longer on ribociclib, and 62.8% of the patients completed the scheduled three years of ribociclib treatment. The Kaplan–Meier curves of the two treatment groups continued to separate with a trend towards widening at 4 years of iDFS: 88.5% vs. 83.6% (HR = 0.715, 95% CI 0.609–0.840, *p* < 0.001), with a 2.7% improvement in iDFS at 3 years and a 4.9% improvement at 4 years [72]. A consistent benefit was shown in the prespecified subgroups. The overall survival data from the trial are currently immature, although a trend towards an OS benefit was shown.

## 3. Comparison of the Results from the NATALEE and monarchE Trials

### 3.1. NATALEE Trial Includes a Wider Population

**Figure 2:**
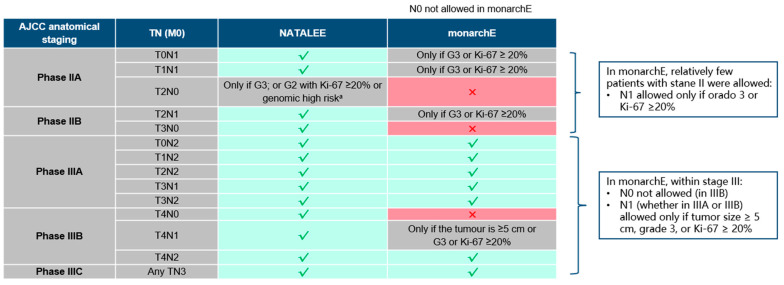
Comparison of NATALEE and monarchE trial inclusion criteria according to TNM staging. A Determined to be high risk by Oncotype DX/Prosigna/MammaPrint/EndoPredict. AJCC, American Joint Committee on Cancer; G, grade; M, metastasis; N, lymph node; T, tumour.

Compared with monarchE, NATALEE included lymph node-negative patients and a broader group of stage II and III patients [73] (Figure 2). In real-world studies, the population meeting the criteria for ribociclib treatment accounted for 41.3–42.9% of HR+/HER2- early-stage breast cancers, the population meeting the criteria for abemaciclib treatment accounted for 17.5–18.1%, and the NATALEE study enrolled larger T1N1, T2N0, and T2N1 populations relative to monarchE. The NATALEE study population was more than double that of monarchE [74,75]. The data from SEER showed that NATALEE included 43% of non-metastatic, hormone receptor-positive, HER2-negative patients, whereas monarchE included only 25% of patients. The T2N0 population (no such patients were included in monarchE; some patients were eligible for NATALEE) accounted for all the non-metastatic, hormone receptor-positive, HER2-negative 16% of patients, whereas the T2N1 population (some patients included in monarchE; all patients were NATALEE-eligible) accounted for 9% of all non-metastatic, hormone-receptor-positive, HER2-negative patients. These are the two most common categories of patients with hormone-receptor-positive, HER2-negative stage II to III breast cancer [76]. The NATALEE results confirm for the first time an iDFS benefit from adjuvant ribociclib in this broader population, opening up a large potential population of eligible patients. By comparing the baselines of the two trials, the NATALEE study included a relatively larger phase II (39.7% vs. 25.4%) and N0 population (27% vs. 0.2%) (Table 2).

The NATALEE and monarchE trials mainly selected the enrolled population based on the TNM stage, histological stage, and Ki67 level among the clinical pathological characteristics. It is worth noting that the T2N0 and G2 population with a high genomic risk can also be included in the NATALEE trial. Combining the previously mentioned risk prediction models and genomic prognostic analysis suggests that identifying the population that will benefit from CDK4/6 inhibitors from more clinical pathological factors and genomic prognostic analysis is a future research direction.

Following the publication of the results of the monarchE trial, the data from real-world studies showed that the monarchE clinicopathological high-risk criteria were associated with an increased risk of recurrence and all-cause mortality in patients with HR+/HER2- early-stage breast cancer. The risk of IDFS, DRFS, and OS events in the high-risk group meeting the monarchE criteria was approximately three times higher than in the low-risk group. The high-risk group had a 5-year iDFS rate of 75.2%, a 5-year mortality rate of 11.2–16.5%, a 5-year iDFS rate of 57.0%, a 10-year mortality rate of 29.1–36.6%, and a 10-year breast-cancer-specific mortality rate of 27.7%, compared with 5-year mortality rates for patients who did not meet the criteria of 7.0% (Stage II-III and lymph node positive) and 2.8% (Stage I or lymph node-negative), and 10-year mortality rates of 24.9% (intermediate-risk, St. Gallen criteria) and 15.9% (low-risk, St. Gallen criteria) [77,78,79,80,81]. Patients meeting the criteria for adjuvant therapy with ribociclib had a 5-year DFS of 86% versus 95% and a 10-year DFS of 77% versus 88%, respectively, and the 5-year OS was 91% and 96% for patients meeting the criteria for adjuvant therapy with bociclib and 78% and 90% for patients not meeting the criteria, respectively. These real-world findings suggest that the inclusion criteria for the monarchE and NATALEE trials are consistent with real-world patient populations at a high risk of recurrence and death [77]. Intriguingly, in a prospective post hoc analysis of the monarchE and NATALEE trial inclusion criteria populations, for stage IIA patients, the prognosis for patients with N0G3 was worse than that for the N1 population, suggesting that for some populations, histological grade 3 has a greater prognostic impact than N0 to N1 [82]. The important impact of histological grade 3 is also reported in a separate article [79]. At this stage, the choice of CDK4/6 inhibitor therapy needs to be based on TNM staging in combination with the clinicopathological features.

### 3.2. Simplified Decision Making for the Surgical Management of Axillary Lymph Nodes

The eligibility for abemaciclib requires meeting the criteria of ≥4 positive axillary lymph nodes, or 1–3 positive axillary lymph nodes with at least one of the following: histological grade 3 and tumour size ≥5 cm. Given the importance of the extent of lymph node disease in these studies, the surgical treatment of the axilla has become a frequently discussed issue, and specifically, do these patients require axillary lymph node dissection (ALND) to determine lymph node burden in order to inform the systemic drug therapy recommendations? More modern studies including ACOSOG Z0011 [83], AMAROS [83,84,85], and the SENOMAC trial [86] have shown that ALND does not improve survival. If patients are found to have 1–3 positive sentinel lymph nodes (SLNs) but do not have the other characteristics of high risk as defined by monarchE, academics have largely discouraged ALND to determine the exact lymph node burden [87]. A retrospective study by the National Cancer Database showed that for patients with 1–2+ SLNs without high risk factors (tumour ≥ 5 cm, grade 3 grade or Ki-67 ≥ 20%), the likelihood of finding ≥ 4+ LNs at the time of ALND, and thus meeting the criteria for adjuvant therapy with abemaciclib, was only 13% [88]. A single-centre retrospective data from France showed that approximately 1000 ALNDs were needed to detect 120 N2 cases and prevent four recurrences [89], and a retrospective study from the UK showed similar results [90]. The Italian National Association of Breast Surgeons (ANISC) believes that in patients with HR+HER2-/cN0-pN1 (sn) breast cancer treated with breast-conserving therapy, the preoperative work-up should be optimised to allow for a more detailed assessment of the axilla and optimise the technique of Sentinel Lymph Node Biopsy (SLNB), and that, if the surgeon deems it appropriate, routine ALND should not be considered but based on the eligibility criteria of the RxPONDER and monarchE trials to determine the treatment recommendations [91]. Post hoc analyses of the SENOMAC trial showed that ALND was required in 104 patients to avoid a single aggressive disease-free survival event 5 years after adjuvant treatment with abemaciclib and resulted in severe or very severe arm dysfunction 1 year postoperatively in nine patients. ALND for the purpose of meeting the criteria for the use of abemaciclib is therefore discouraged [92]. With the approval of ribociclib, this issue will be perfected and all lymph node-positive patients will be eligible for ribociclib, which is important for simplifying the treatment decisions.

### 3.3. Similar iDFS Benefit of Ribociclib and Abemaciclib

**Figure 3:**
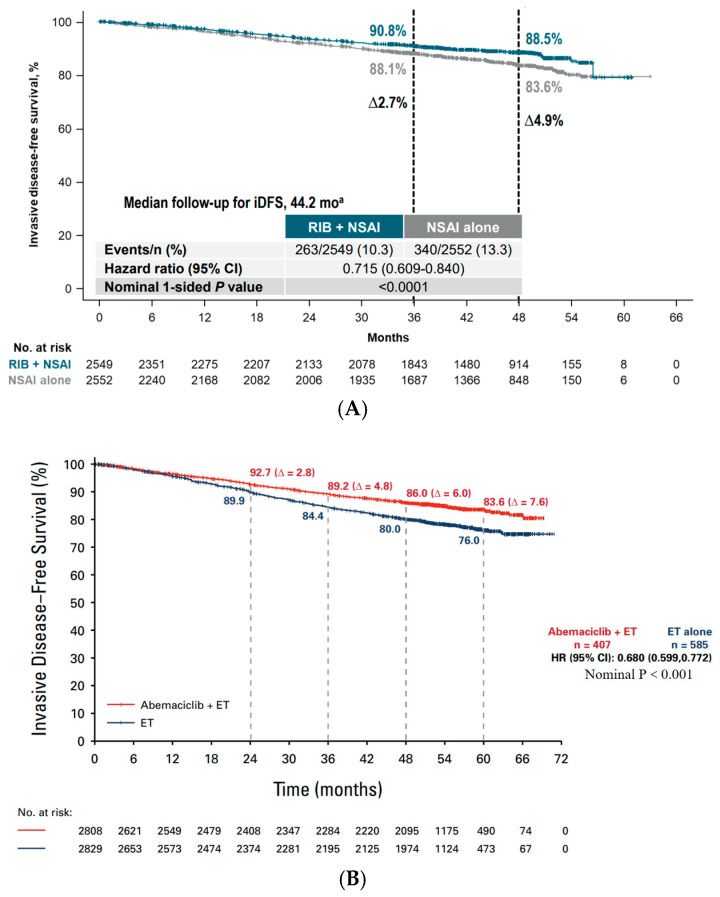
Kaplan–Meier plots of NATALEE (**A**) and monarchE (**B**).

The two positive clinical trials, NATALEE and monarchE, reported results at multiple time points (Figure 3), and Table 3 compares the most recent data from the two trials. iDFS, the primary endpoint, showed a durable and stable iDFS benefit, with an absolute benefit continuing to increase with a longer follow-up; the Kaplan–Meier curves continued to separate and widen, and there was no overlap or crossover similar to that of the PENELOPE-B trial which showed an overlap or crossover after stopping treatment. monarchE included a relatively higher-risk population, which reflected a worse prognosis in terms of absolute iDFS. Comparable efficacy benefits were observed in the risk ratios for the primary endpoint, iDFS, the secondary endpoint, DDFS/DRFS, and OS.

When comparing the efficacy benefits, it is important to take into account the difference in the control groups in the two trials. In the monarchE trial, approximately 30% of the enrolled population used TAM as their first endocrine treatment of choice, and subgroup analyses showed an iDFS HR = 0.738 (95% CI 0.634, 0.859) in the AI subgroup and an iDFS HR = 0.561 (95% CI 0.445, 0.708), suggesting that the treatment benefit in the monarchE study was driven more by the TAM subgroup, and that there was undertreatment of high-risk populations with TAM treatment [70].

### 3.4. Safety Profiles of Ribociclib and Abemaciclib: Management of Toxicity

Overall, ribociclib and abemaciclib were tolerated and had manageable adverse events in early breast cancer. In separate clinical trials, 62.8% of the patients completed three years of ribociclib treatment and 69% completed two years of abemaciclib treatment. The incidence of adverse events for both drugs was collated from the information concerning the prescribing of the two drugs [93,94].

**Figure 4:**
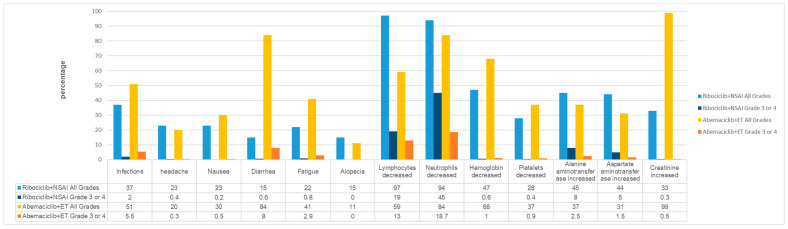
Adverse reaction comparisons in NATALEE and monarchE.

Although direct comparisons between the trials could not be made, higher rates of neutropenia were reported with ribociclib (45% grade 3 neutropenia with ribociclib in the NATALEE trial; 18.7% grade 3 neutropenia with abemaciclib in the monarchE trial). Diarrhoea was reported more frequently with abemaciclib (84% for any grade of diarrhoea and 8% for grade 3 or greater diarrhoea with abemaciclib in monarchE; 15% for any grade of diarrhoea and <1% for grade 3 or greater diarrhoea with ribociclib in NATALEE) (Figure 4). Ribociclib’s liver-related adverse events are of concern, with 8% of patients treated with NATALEE experiencing liver-related adverse events and 5% experiencing QTc prolongation. Of note, the dose of ribociclib was reduced from 600 mg in advanced breast cancer to 400 mg in early breast cancer, which also resulted in a relative reduction in the incidence of QT interval prolongation and haematological toxicity being observed.

Ribociclib and abemaciclib can be managed by dose adjustment for adverse events. In early breast cancer, ribociclib allows a single dose reduction from 400 mg qd to 200 mg qd and abemaciclib allows two dose reductions, from 150 mg bid to 100 mg bid, or a further reduction to 50 mg bid. The general dose adjustment principles for ribociclib in early breast cancer are as follows: For Grade 1 or 2 adverse events, no dose adjustment is required and appropriate medical therapy and monitoring should be initiated as clinically indicated. For Grade 3 adverse events, dose interruption should be performed until recovery to Grade < 1 followed by the resumption of ribociclib at the same dose level. If Grade 3 recurs, ribociclib should be resumed at the next lower dose level. For Grade 4 adverse events, ribociclib should be discontinued. For QT interval prolongation, hepatobiliary toxicity, and neutropenia, dose adjustments are required in accordance with the instructions. Abemaciclib-induced diarrhoea is managed on a graded scale, with no dose adjustment required for Grade 1 diarrhoea; if Grade 2 diarrhoea does not resolve within 24 h to ≤Grade 1, doses should be suspended until resolution, and no dose reduction is required. Grade 3 and 4 diarrhoea require dose suspension until the toxicity resolves to ≤Grade 1 with resumption at the next lower dose.

### 3.5. Similar Patient-Reported Outcomes but Patients Preferred Ribociclib More

In the analyses of the prespecified health-related quality of life endpoints in the NATALEE and monarchE trials, the results showed that the addition of ribociclib or abemaciclib to endocrine therapy did not have an impact on the quality of life. Direct comparisons were made more difficult by the different scales chosen for the two studies, with NATALEE using the EORTC QLQ-C30, EORTC QLQ-BR23, EQ-5D-5L, and HADS scales, and monarchE using the FACT-B, FACT-ES, and FACIT-Fatigue scales. In the NATALEE trial, physical functioning was maintained with the addition of ribociclib to standard-of-care NSAI. The global health status was not impacted over time in both arms [95]. In the monarchE trial, PROs were similar between the arms, including being “bothered by side-effects of treatment”, except for diarrhoea. At ≥3 months, most patients reporting diarrhoea reported “a little bit” or “somewhat”. Adjuvant abemaciclib + ET has an acceptable safety profile and tolerability as supported by PRO findings. Most AEs were reversible and manageable with comedications and/or dose modifications, consistent with the known abemaciclib toxicity profile [96]. Longer follow-up results showed similar PROs to the baseline in both groups during the post-treatment follow-up [97]. Of note, in monarchE, most patients had grade 2/3 events within the first 3 months with a median duration of ≤7 days, while the first PRO assessment was collected at 3 months, but the available data may not clearly demonstrate the impact of diarrhoea on patients’ quality of life.

In addition, a web-based discrete choice experiment survey was conducted among patients with EBC between January and May 2023 before the NATALEE results were available. The patient preferences for attributes that significantly influence treatment decisions were, in descending order, higher efficacy (iDFS), lower risk of diarrhoea, lower risk of fatigue, shorter duration of treatment, and lower risk of venous thromboembolic events. Patients with HR+/HER2- early-stage breast cancer had a strong preference for a lower risk of AEs, and therefore preferred treatment regimens that were more similar to those that were received with the clinical experience of similar ribociclib treatment regimens. These patient preferences are important for shared decision making when discussing the addition of CDK4/6 inhibitors to adjuvant therapy for eligible HR+/HER2- early breast cancer patients [98].

### 3.6. Regulatory Considerations of CDK4/6 Inhibitors for Adjuvant Therapy

With the results of the primary analysis of monarchE, abemaciclib was first approved for the adjuvant treatment of early-stage breast cancer in October 2021, with the population limited to those with Ki-67 ≥ 20% in Cohort 1 [99]. Prior to this, the Ki-67 threshold was not used as a qualifier for the indication of antitumor drugs in breast cancer. The main consideration was that at the time of the first OS interim analysis (cut-off 1 April 2021), most patients had completed their adjuvant therapy but showed a trend towards potentially impaired OS in the ITT population (HR = 1.091, [95% CI 0.818–1.455]), and so the FDA limited the indication to the Ki-67 ≥ 20% population in Cohort 1 [100]. OS impairment was no longer observed in the ITT population until the second interim OS analysis (cut-off 1 July 2022) (OS HR = 0.929; [95% CI 0.748 to 1.153]), and the FDA did not approve the entire population in Cohort 1 until 3 March 2023 [101]. The main consideration for limiting the indication to Cohort 1 was that Cohort 2 (approximately 9% of the total population) started enrolment approximately 12 months after Cohort 1, and exploratory analyses showed an imbalance in the number of deaths (10 deaths in the abemaciclib plus ET group and 5 deaths in the ET-only group) and in favour of the ET-only group. As the majority of patients (91%) and events (95%) in the second OS interim analysis were from Cohort 1, the FDA review panel concluded that the favourable treatment effect noted in the IDFS in the ITT population was largely attributable to patients in Cohort 1. Therefore, while the updated indication expands the Cohort 1 population by removing the Ki-67 ≥ 20% requirement, it still excludes high-risk patients as defined in Cohort 2 [69]. In the most recent update of the five-year follow-up results (cut-off 3 July 2023), a total of 22 OS events occurred in both groups in Cohort 2 (11 vs. 11), and a trend towards impaired OS was still observed (OS HR = 1.078; [95% CI 0.465 to 2.501]), with a longer follow-up yet to observe the maturing outcome of OS in Cohort 2 [70].

In September 2024, based on the results of the NATALEE trial, ribociclib was approved for patients with any lymph node involvement (excluding microscopic nodal involvement), or if there was no nodal involvement, either a tumour size > 5 cm, or tumour size 2 to 5 cm with either Grade 2 (and high genomic risk or Ki67 ≥ 20%) or Grade 3. This approval is based on the results of the trial’s primary analyses, including the entire population of the NATALEE trial inclusion criteria. At the time of the primary analysis (cut-off 11 January 2023), a statistically significant improvement in iDFS was observed in the intention-to-treat patient population, with a 36-month iDFS of 90.7% versus 87.6% and an iDFS HR = 0.749 (95% CI 0.628, 0.892). Consistent results were shown across the subgroups, both in the stage II subgroup (iDFS HR = 0.76; [95% CI 0.53 to 1.10]) or stage III subgroup (iDFS HR = 0.74; [95% CI 0.59 to 0.93]), and in the N0 subgroup (iDFS HR = 0.63; [95% CI 0.34 to 1.16]) or the N1-N3 subgroup (iDFS HR = 0.77; [95% CI 0.63 to 0.94]). A trend towards an OS benefit was demonstrated at the first interim analysis of OS, OS HR = 0.76 (95% CI 0.54, 1.07) [22]. The subgroup analyses and OS results were continued in the final iDFS analysis and four-year follow-up results [71,72].

The drug approval process fully reflects the rigorous review of clinical data by the regulatory agencies, emphasising a decision-making process that is centred on patient safety and treatment efficacy. Historically, ribociclib expanded the population of indications for adjuvant therapy with CDK4/6 inhibitors, but based on the results of adequate subgroup analyses and positive OS data, ribociclib was able to be approved for the full population at the time of first approval.

### 3.7. Limitations of CDK4/6 Inhibitor Adjuvant Therapy and Unanswered Clinical Questions

Both the NATALEE and monarchE studies were open-label designs, and a common problem is that imbalances in early deletion from a trial can invalidate randomisation if the patients who drop out of the trial are different from those who remain in the trial. A high degree of imbalance in early censoring may be caused by drug toxicity. It has been reported in the literature that the trial group of the monarchE study had more censoring in iDFS and OS, and that the iDFS benefit may have been caused by informative censoring [102]. While the NATALEE control group had more censoring, it is possible that analyses using the reverse KM method may have yielded different results.

Although both the NATALEE and the MonarchE trials have shown significant benefits of iDFS, the follow-up time is currently relatively short, and the OS data are not yet mature. Long-term follow-up is still needed to evaluate the impact of adjuvant treatment with CDK4/6 inhibitors on OS and late toxicity. Only patients with Ki67 ≥ 20% were selected as a single high-risk clinical pathological feature in the MonarchE trial Cohort 2. The treatment benefits, especially the OS benefits, of this group of people are still unclear. For the relatively low-risk stage IIA T2N0 population in the NATALEE trial, it is not yet possible to fully determine the entire population that can benefit from ribociclib treatment based on G3, Ki67 ≥ 20%, or genomic high risk. Due to these gaps in evidence, additional research evidence for a specific high-risk population is needed in the future. In addition, the optimal duration of treatment with CDK4/6 inhibitors is not yet clear, and it is still unknown whether prolonging or shortening the duration of CDK4/6 inhibitor treatment will affect the long-term efficacy and safety outcomes. When drug switching is required due to intolerable adverse events with one of the CDK4/6 inhibitors, the timing of drug switching and the duration of treatment after drug switching are also unclear. Additional efficacy and safety data are still needed to guide the clinical practice in the adjuvant setting in combination with other targeted agents or immune checkpoint inhibitors.

## 4. Research Directions for CDK4/6 Inhibitors in Adjuvant Therapy

### 4.1. Combinations or Sequential Therapies with PARP Inhibitors or Immunotherapy

For patients with pathogenic variants of the genes BRCA1/BRCA2, the OlympiA trial achieved positive results in a high-risk population of hormone receptor-positive, HER2-negative breast cancers and variants of the genes BRCA1/BRCA2 [58]. For the treatment selection of the population with an overlap between OlympiA and monarchE or NATALEE, taking into account the difficulty in tolerating the combination of the two drugs’ overlapping adverse effects, sequential therapy may be considered, and there are no data on the sequential use of CDK4/6 inhibitors after olaparib. However, given that monarchE permits the initiation of adjuvant abemaciclib within 16 months of definitive surgery for breast cancer and NATALEE permits the initiation of adjuvant ribociclib within 12 months of the initiation of endocrinology, sequential therapies are not out of the question for high-risk patients.

Data on the combination with PD-1 in the adjuvant phase have not been reported, and the latest CheckMate-7FL [103] and KEYNOTE-756 trials [104] showed that the addition of PD-1 in high-risk ER+ /HER2- early-stage breast cancers significantly improved the pCR of neoadjuvant therapy. In the KEYNOTE-756 trial, the adjuvant phase of treatment would have received six months of pembrolizumab treatment, with the combination endocrine therapy to be determined by the investigator and may include abemaciclib. In the CheckMate-7FL trial, with abemaciclib approved for adjuvant therapy, the protocol was revised in April 2022, with the primary endpoint focusing only on pCR. EFS was changed from the primary endpoint to an exploratory endpoint, with new enrolment stopped, and with a shorter duration of follow-up to 1 year postoperatively, and adjuvant therapy was changed to open-label. Considering that the combination of PD-1 and CDK4/6 inhibitors increased the intolerable immune-related liver toxicity in previous neoadjuvant or late-stage studies, clinical development was stopped at the phase I stage [105,106,107]. More safety data are still needed to analyse the possibility of combining PD-1 and CDK4/6 inhibitors. Longer follow-up of the KEYNOTE-756 trial is needed to clarify the efficacy of the PD-1 sequential CDK4/6 inhibitor.

### 4.2. Other Ongoing Clinical Trials in Adjuvant Therapy for HR+/HER2- Early Breast Cancer

There are two ongoing phase III studies of CDK4/6 inhibitors applied to the adjuvant treatment of HR+/HER2- early breast cancer, dalpiciclib (SHR6390-III-303, NCT04842617) and TQB3616 (TQB3616-III-03, NCT05780567). The enrolment criteria for these two studies are generally similar to those of monarchE but slightly different.

Palbociclib still continues to be explored for its efficacy in adjuvant therapy in a population limited to isolated locally recurrent breast cancer after the completion of local therapy, (POLAR, NCT03820830). Abemaciclib has been further explored for adjuvant efficacy in a wider population with intermediate to high clinical or genomic risk including c/ypN0-1 (ADAPTlate, NCT04565054).

Several phase III trials are currently investigating the efficacy of SERDs in EBC, including ELEGANT for elacestrant (NCT06492616), EMBER-4 for imlunestrant (NCT05514054), lidERA for giredestrant (NCT04961996), and CAMBRIA-1 (NCT05774951) and CAMBRIA-2 (NCT05952557) for camizestrant. These are ongoing trials and the results have not yet been reported (Table 4).

In the last three years, abemaciclib and ribociclib have remained the main choices for intensive endocrine adjuvant therapy, with ribociclib being the choice for adjuvant therapy in stage II or lymph node-negative patients for a long time to come. As new therapeutic options become available, there is a need for further research into the combination possibilities and interactions between the different regimens.

### 4.3. Prognostic Value and Monitoring Implications of ctDNA Testing

Circulating tumour DNA (ctDNA) was prospectively tested as a powerful biomarker prior to macroscopic recurrence in two large trials, PENELOPE-B and monarchE. In PENELOPE-B, baseline ctDNA detection 9% (7/78) patients, iDFS and DDFS were significantly worse in the ctDNA subgroup detected at the baseline than in the undetected subgroup. However, the sensitivity of ctDNA to recurrence was not perfect, especially for late recurrences. Therefore the application of ctDNA is not recommended as a criterion to qualify for the application of CDK4/6 inhibitors [108]. Analysis of ctDNA detection was performed in the monarchE trial. In a patient subset from monarchE enriched for IDFS events, ctDNA detection was relatively infrequent (<20%); however, its detection at any time during the 24 months of the study’s therapy was adversely prognostic. As compared to patients who had remaining ctDNA positive (+) results, patients who had clearance of ctDNA were more likely to have a positive ctDNA test. Patients who had clearance of ctDNA on therapy had a lower risk of IDFS events, but the event risk still remained clinically meaningful in these patients [109].

The detection of ctDNA in the post-treatment surveillance setting can identify molecular recurrence before it becomes apparent, but there are still many challenges, including low ctDNA levels and detection rates in early-stage breast cancer, unknown optimal frequency and time intervals for testing, and the possibility that metastatic cells from ER-positive tumours may be dormant in non-bloodstream regions such as the bone marrow. There are a few prospective trials examining this area, including LEADER (NCT03285412), DARE (NCT04567420), and TREAT ctDNA (NCT05512364).

## 5. Conclusions

Following the approval of abemaciclib and ribociclib for early-stage HR+/HER2- breast cancer, CDK4/6 inhibitors have become integral to adjuvant therapy. Adding a CDK4/6 inhibitor to systemic and endocrine therapy may further benefit high-risk patients. Ribociclib and abemaciclib show comparable efficacy and manageable safety profiles, with ribociclib offering broader applicability and simplifying the decisions related to axillary lymph node surgery, making it a preferred option in high-risk populations.

However, key questions remain. Ongoing trials are needed to explore the optimal treatment sequencing, combination strategies with immunotherapy or PARP inhibitors, and the role of biomarkers in patient selection. Additionally, the long-term effects of CDK4/6 inhibitors on survival, resistance, and late-onset adverse events must be better understood. Continued research, especially in diverse, international cohorts, will be crucial to refining the treatment approaches and improving patient outcomes.

## Figures and Tables

**Table 1:** Clinical trials of three CDK4/6 inhibitors performing adjuvant therapy.

	PALLAS [19]	PENELOPE B [20]	monarchE [21,68,69,70]	NATALEE [22,71,72]
CDK4/6 inhibitor	Palbociclib	Palbociclib	Abemaciclib	Ribociclib
Sample size, N	5796	1250	5637	5101
Population	stage II-III	High risk after neoadjuvant chemotherapy	clinical high-risk patients	stage II–III
dosage	125 mg qd 3 weeks on/1 week off	125 mg qd 3 weeks on/1 week off	150 mg bid continuous	400 mg qd 3 weeks on/1 week off
Duration of medication	2 years	1 year	2 years	3 years
Primary endpoint	iDFS	iDFS	iDFS	iDFS
ET	AI or TAM ±LHRH agonist	AI or TAM ±LHRH agonist	AI or TAM ±LHRH agonist	AI ±LHRH agonist
iDFS	4 years of iDFS: 84.2% vs. 84.5% (HR = 0.96, 95% CI 0.81–1.14, *p* = 0.65)	4 years of iDFS: 73% vs. 72.4% (HR = 0.93, 95% CI 0.74–1.17, *p* = 0.53)	5 years of DFS: 83.6% vs. 79.4% (HR = 0.68, 95% CI 0.599–0.772, *p* < 0.001)	4 years of iDFS: 88.5% vs. 83.6% (HR = 0.715, 95% CI 0.609–0.840, *p* < 0.001)
Result	negative	negative	positive	positive

AI, aromatase inhibitor; ET, endocrine therapy; iDFS, invasive disease-free survival; LHRH, luteinising hormone-releasing hormone; HR, hazard; LHRH, luteinising hormone-releasing hormone; HR, hazard ratio; TAM, tamoxifen.

**Table 2:** NATALEE and monarchE trial enrolment population baseline.

	NATALEE		monarchE	
	Ribociclib + NSAI	NSAI Alone	Abemaciclib + ET	ET Alone
Median age (range), years	52 (24–90)	52 (24–89)	51 (23–89)	51 (22–86)
Pre-menopausal rate	44%	44%	43.5%	43.5%
Percentage of males	0.4%	0.4%	0.7%	0.5%
Anatomical Staging				
I	0.4%	0.2%	0.1%	0.0%
IIA	18.8%	20.4%	11.5%	12.5%
IIB	20.9%	20.1%	13.9%	13.7%
III	59.9%	59.2%	74.1%	73.4%
Lymph node status				
N0	27%	29%	0.2%	0.2%
N1	41%	41%	40%	40%
N2-3	19%	18%	60%	59%
Histologic grade at diagnosis				
Grade 1	8.6	9.4	7.4	7.6
Grade 2	57.2	56.9	48.9	49.3
Grade 3	20.4	21.5	38.8	37.7
Grade cannot be assessed	11.5	10.1	4.5	4.9
Previous chemotherapy				
Any	88.2%	88.0%	95.5%	95.3%
Neoadjuvant chemotherapy	42.6%	42.9%	37.0%	37.0%
Adjuvant chemotherapy	48.0%	47.8%	58.5%	58.2%

Data are presented as no. (%) unless otherwise noted. Where values do not add up to 100%, remaining data are missing, unavailable, or could not be assessed.

**Table 3:** Summary of NATALEE and monarchE efficacy results.

	NATALEE [72]	monarchE [70]
follow-up time	44.2 months	54 months
iDFS	HR = 0.715 (95% CI 0.609–0.8406)	HR = 0.680 (95% CI 0.599–0.772)
3-year iDFS rate	90.8% vs. 88.1%	89.2% vs. 84.4%
	Δ2.7%	Δ4.8%
4-year iDFS rate	88.5% vs. 83.6%	86.0% vs. 80.0%
	Δ4.9%	Δ6.0%
5-year iDFS rate	-	83.6% vs. 76.0%
	-	Δ7.6%
DDFS/DRFS	0.715 (95% CI 0.604–0.847)	0.675 (95% CI 0.588–0.774)
OS	0.827 (95% CI 0.636–1.074)	0.903 (95% CI 0.749–1.088)

DDFS, distant disease-free survival; DRFS, distant relapse-free survival; HR, hazard ratio; IDFS, invasive disease-free survival; OS, overall survival. IDFS, invasive disease-free survival; OS, overall survival.

**Table 4:** Completed and ongoing trials on adjuvant endocrine therapy in HR+/HER2- EBC.

Trial	Drug	Enrolment	Inclusion Criteria	Primary Endpoint	NCT Number
NATALEE	Ribociclib	5101	stage II-III	IDFS	NCT03701334
monarchE	Abemaciclib	5637	clinical high-risk node positive	IDFS	NCT03155997
SHR6390-III-303	Dalpiciclib	5274	clinical high-risk node positive	IDFS	NCT04842617
TQB3616-III-03	TQB3616	1946	clinical high-risk node positive	IDFS	NCT05780567
POLAR	Palbociclib	400	isolated locoregional recurrence	IDFS	NCT03820830
ADAPTlate	Abemaciclib	1260	high clinical or genomic risk after 1–6 yrs adjuvant AI	IDFS	NCT04565054
ELEGANT	Elacestrant	4220	high risk of recurrence node positive	IBCFS	NCT06492616
EMBER-4	imlunestrant	6000	post 2–5 yrs adj. AI ± CDK4/6i	IDFS	NCT05514054
lidERA	giredestrant	4100	medium- and high-risk Stage I-III	IDFS *	NCT04961996
lidERA substudy	giredestrant + abemaciclib	100	medium- and high-risk Stage I-III	IDFS *	NCT04961996
CAMBRIA-1	camizestrant	4300	medium- and high-risk post 2–5 yrs adj. AI ± CDK4/6i	IBCFS	NCT05774951
CAMBRIA-2	camizestrant + abemaciclib	5500	high-intermediate to high risk	IBCFS	NCT05952557

* Excluding second primary non-breast cancers.

## References

[1] Giaquinto A.N., Sung H., Newman L.A., Freedman R.A., Smith R.A., Star J., Jemal A., Siegel R.L. (2024). Breast cancer statistics 2024. CA Cancer J. Clin..

[2] Howlader N., Altekruse S.F., Li C.I., Chen V.W., Clarke C.A., Ries L.A., Cronin K.A. (2014). US Incidence of Breast Cancer Subtypes Defined by Joint Hormone Receptor and HER2 Status. J. Natl. Cancer Inst..

[3] Burstein H.J. (2020). Systemic Therapy for Estrogen Receptor-Positive, HER2-Negative Breast Cancer. N. Engl. J. Med..

[4] National Comprehensive Cancer Network (2024). Breast Cancer. Version 4. http://www.nccn.org/professionals/physician_gls/pdf/breast.pdf.

[5] Loibl S., André F., Bachelot T., Barrios C., Bergh J., Burstein H., Cardoso M., Carey L., Dawood S., Del Mastro L. (2023). Early breast cancer: ESMO Clinical Practice Guideline for diagnosis, treatment and follow-up. Ann. Oncol..

[6] Pan H., Gray R., Braybrooke J., Davies C., Taylor C., McGale P., Peto R., Pritchard K.I., Bergh J., Dowsett M. (2017). 20-Year Risks of Breast-Cancer Recurrence after Stopping Endocrine Therapy at 5 Years. N. Engl. J. Med..

[7] O’Sullivan C.C., Clarke R., Goetz M.P., Robertson J. (2023). Cyclin-Dependent Kinase 4/6 Inhibitors for Treatment of Hormone Receptor-Positive, ERBB2-Negative Breast Cancer: A Review. JAMA Oncol..

[8] Hortobagyi G.N., Stemmer S.M., Burris H.A., Yap Y.-S., Sonke G.S., Paluch-Shimon S., Campone M., Blackwell K.L., André F., Winer E.P. (2016). Ribociclib as First-Line Therapy for HR-Positive, Advanced Breast Cancer. N. Engl. J. Med..

[9] Slamon D.J., Neven P., Chia S., Fasching P.A., Laurentiis M.D., Im S.-A., Petrakova L., Bianchi G.B., Estenva F.J., Martin M. (2018). Phase III randomized study of ribociclib and fulvestrant in hormone receptor-positive, human epidermal growth factor receptor 2-negative advanced breast cancer: Monaleza-3. J. Clin. Oncol..

[10] Tripathy D., Im S.-A., Colleoni M., Franke F., Bardia A., Harbeck N., Hurvitz S.A., Chow L., Sohn J., Lee K.S. (2018). Ribociclib plus endocrine therapy for premenopausal women with hormone-receptor-positive, advanced breast cancer (MONALEESA-7): A randomised phase 3 trial. Lancet Oncol..

[11] Sledge G.W., Toi M., Neven P., Sohn J., Inoue K., Pivot X., Burdaeva O., Bkera M., Basuda N., Kaufman P.A. (2017). MONARCH 2: Abemaciclib in combination with fulvestrant in women with HR+/HER2- advanced breast cancer who had progressed while receiving endocrine therapy. J. Clin. Oncol..

[12] Goetz M.P., Toi M., Campone M., Sohn J., Paluch-Shimon S., Huober J., Park I.H., Trédan O., Chen S.-C., Manso L. (2017). MONARCH 3: Abemaciclib As Initial Therapy for Advanced Breast Cancer. J. Clin. Oncol..

[13] Turner N.C., Ro J., André F., Loi S., Verma S., Iwata H., Harbeck N., Loibl S., Bartlett C.H., Zhang K. (2015). Palbociclib in Hormone-Receptor–Positive Advanced Breast Cancer. N. Engl. J. Med..

[14] Finn R.S., Martin M., Rugo H.S., Jones S., Im S.-A., Gelmon K., Harbeck N., Lipatov O.N., Walshe J.M., Moulder S. (2016). Palbociclib and Letrozole in Advanced Breast Cancer. N. Engl. J. Med..

[15] Hortobagyi G.N., Stemmer S.M., Burris H.A., Yap Y.-S., Sonke G.S., Hart L., Campone M., Petrakova K., Winer E.P., Janni W. (2022). Overall Survival with Ribociclib plus Letrozole in Advanced Breast Cancer. N. Engl. J. Med..

[16] Slamon D.J., Neven P., Chia S., Fasching P.A., De Laurentiis M., Im S.-A., Petrakova K., Bianchi G.V., Esteva F.J., Martín M. (2020). Overall Survival with Ribociclib plus Fulvestrant in Advanced Breast Cancer. N. Engl. J. Med..

[17] Im S.-A., Lu Y.-S., Bardia A., Harbeck N., Colleoni M., Franke F., Chow L., Sohn J., Lee K.-S., Campos-Gomez S. (2019). Overall Survival with Ribociclib plus Endocrine Therapy in Breast Cancer. N. Engl. J. Med..

[18] Sledge G.W., Toi M., Neven P., Sohn J., Inoue K., Pivot X., Burdaeva O., Okera M., Masuda N., Kaufman P.A. (2020). The effect of abemaciclib plus fulvestrant on overall survival in hormone receptor-positive, ERBB2-negative breast cancer that progressed on endocrine therapy–MONARCH 2: A randomized clinical trial. JAMA Oncol..

[19] Gnant M., Dueck A.C., Frantal S., Martin M., Burstein H.J., Greil R., Fox P., Wolff A.C., Chan A., Winer E.P. (2022). Adjuvant palbociclib for early breast cancer: The PALLAS trial results (ABCSG-42/AFT-05/BIG-14–03). J. Clin. Oncol..

[20] Loibl S., Marmé F., Martin M., Untch M., Bonnefoi H., Kim S.-B., Bear H., McCarthy N., Olivé M.M., Gelmon K. (2021). Palbociclib for Residual High-Risk Invasive HR-Positive and HER2-Negative Early Breast Cancer—The Penelope-B Trial. J. Clin. Oncol..

[21] Johnston S.R.D., Harbeck N., Hegg R., Toi M., Martin M., Shao Z.M., Zhang Q.Y., Rodriguez J.L.M., Campone M., Hamilton E. (2020). Abemaciclib Combined With Endocrine Therapy for the Adjuvant Treatment of HR+, HER2−, Node-Positive, High-Risk, Early Breast Cancer (monarchE). J. Clin. Oncol..

[22] Slamon D., Lipatov O., Nowecki Z., McAndrew N., Kukielka-Budny B., Stroyakovskiy D., Yardley D.A., Huang C.-S., Fasching P.A., Crown J. (2024). Ribociclib plus Endocrine Therapy in Early Breast Cancer. N. Engl. J. Med..

[23] Lowery A., Kell M., Glynn R. (2012). Locoregional recurrence after breast cancer surgery: A systematic review by receptor phenotype. Breast Cancer Res. Treat..

[24] Mariotto A.B., Zou Z., Zhang F., Howlader N., Kurian A.W., Etzioni R. (2018). Can We Use Survival Data from Cancer Registries to Learn about Disease Recurrence? The Case of Breast Cancer. Cancer Epidemiol. Biomark. Prev..

[25] Taylor C., McGale P., Probert J., Broggio J., Charman J., Darby S.C., Kerr A.J., Whelan T., Cutter D.J., Mannu G. (2023). Breast cancer mortality in 500 000 women with early invasive breast cancer diagnosed in England, 1993–2015: Population based observational cohort study. BMJ.

[26] Early Breast Cancer Trialists’ Collaborative Group (EBCTCG) (2005). Effects of chemotherapy and hormonal therapy for early breast cancer on recurrence and 15-year survival: An overview of the randomised trials. Lancet.

[27] Loibl S., Poortmans P., Morrow M., Denkert C., Curigliano G. (2021). Breast cancer. Lancet.

[28] Garutti M., Griguolo G., Botticelli A., Buzzatti G., De Angelis C., Gerratana L., Molinelli C., Adamo V., Bianchini G., Biganzoli L. (2022). Definition of High-Risk Early Hormone-Positive HER2− Negative Breast Cancer: A Consensus Review. Cancers.

[29] Azim H.A., Partridge A.H. (2014). Biology of breast cancer in young women. Breast Cancer Res..

[30] Dialla P.O., Quipourt V., Gentil J., Marilier S., Poillot M.-L., Roignot P., Altwegg T., Darut-Jouve A., Guiu S., Arveus P. (2015). In breast cancer, are treatments and survival the same whatever a patient’s age? a population-based study over the period 1998–2009. Geriatr. Gerontol. Int..

[31] Gabriel N.H., James L.C., Carl J.D., Smith A.B., Johnson C.D., Williams E.F., Brown G.H., Davis I.J., Miller K.L., Wilson M.N., Mahul B.A. (2017). Breast. American Joint Committee on Cancer (AJCC) Cancer Staging Manual.

[32] Sestak I., Dowsett M., Zabaglo L., Lopez-Knowles E., Ferree S., Cowens J.W., Cuzick J. (2013). Factors Predicting Late Recurrence for Estrogen Receptor–Positive Breast Cancer. JNCI J. Natl. Cancer Inst..

[33] Colleoni M., Sun Z., Price K.N., Karlsson P., Forbes J.F., Thürlimann B., Gianni L., Castiglione M., Gelber R.D., Coates A.S. (2016). Annual Hazard Rates of Recurrence for Breast Cancer During 24 Years of Follow-Up: Results From the International Breast Cancer Study Group Trials I to V. J. Clin. Oncol..

[34] Nielsen T.O., Leung S.C.Y., Rimm D.L., Dodson A., Acs B., Badve S., Denkert C., Ellis M.J., Fineberg S., Flowers M. (2021). Assessment of Ki67 in Breast Cancer: Updated Recommendations From the International Ki67 in Breast Cancer Working. J. Natl. Cancer Inst..

[35] Haybittle J.L., Blamey R.W., Elston C.W., Johnson J., Doyle P.J., Campbell F.C., I Nicholson R., Griffiths K. (1982). A prognostic index in primary breast cancer. Br. J. Cancer.

[36] Cuzick J., Dowsett M., Pineda S., Wale C., Salter J., Quinn E., Zabaglo L., Mallon E., Green A.R., Ellis I.O. (2011). Prognostic Value of a Combined Estrogen Receptor, Progesterone Receptor, Ki-67, and Human Epidermal Growth Factor Receptor 2 Immunohistochemical Score and Comparison With the Genomic Health Recurrence Score in Early Breast Cancer. J. Clin. Oncol..

[37] Ravdin P.M., Siminoff L.A., Davis G.J., Bercer M.B., Hewlett J., Gerson N., Parker H.L. (2001). Computer programme to assist in making decisions about adjuvant therapy for women with early breast cancer. J. Clin. Oncol..

[38] Dowsett M., Sestak I., Regan M.M., Dodson A., Viale G., Thürlimann B., Colleoni M., Cuzick J. (2018). Integration of Clinical Variables for the Prediction of Late Distant Recurrence in Patients With Estrogen Receptor–Positive Breast Cancer Treated With 5 Years of Endocrine Therapy: CTS5. J. Clin. Oncol..

[39] Wishart G.C., Azzato E.M., Greenberg D.C., Rashbass J., Kearins O., Lawrence G., Caldas C., Pharoah P.D. (2010). PREDICT: A new UK prognostic model that predicts survival following surgery for invasive breast cancer. Breast Cancer Res..

[40] Sengupta A.K., Gunda A., Malpani S., Serkad C.P.V., Basavaraj C., Bapat A., Bakre M.M. (2020). Comparison of breast cancer prognostic tests CanAssist Breast and Oncotype DX. Cancer Med..

[41] Ma Z., Huang S., Wu X., Huang Y., Chan S.W.-C., Lin Y., Zheng X., Zhu J. (2022). Development of a Prognostic App (iCanPredict) to Predict Survival for Chinese Women With Breast Cancer: Retrospective Study. J. Med Internet Res..

[42] Syed Y.Y. (2020). Oncotype DX Breast Recurrence Score®: A Review of its Use in Early-Stage Breast Cancer. Mol. Diagn. Ther..

[43] Fan C., Oh D.S., Wessels L., Weigelt B., Nuyten D.S., Nobel A.B., Van’T Veer L.J., Perou C.M. (2006). Concordance among Gene-Expression–Based Predictors for Breast Cancer. N. Engl. J. Med..

[44] Filipits M., Rudas M., Jakesz R., Dubsky P., Fitzal F., Singer C.F., Dietze O., Greil R., Jelen A., Sevelda P. (2011). A New Molecular Predictor of Distant Recurrence in ER-Positive, HER2-Negative Breast Cancer Adds Independent Information to Conventional Clinical Risk Factors. Clin. Cancer Res..

[45] Ohnstad H.O., Borgen E., Falk R.S., Lien T.G., Aaserud M., Sveli M.A.T., Kyte J.A., Kristensen V.N., Geitvik G.A., Schlichting E. (2017). Prognostic value of PAM50 and risk of recurrence score in patients with early-stage breast cancer with long-term follow-up. Breast Cancer Res..

[46] Zhang Y., Schnabel C.A., Schroeder B.E., Jerevall P.-L., Jankowitz R.C., Fornander T., Stål O., Brufsky A.M., Sgroi D., Erlander M.G. (2013). Breast Cancer Index Identifies Early-Stage Estrogen Receptor–Positive Breast Cancer Patients at Risk for Early- and Late-Distant Recurrence. Clin. Cancer Res..

[47] Sparano J.A., Gray R.J., Makower D.F., Pritchard K.I., Albain K.S., Hayes D.F., Geyer C.E., Dees E.C., Goetz M.P., Olson J.A. (2018). Adjuvant Chemotherapy Guided by a 21-Gene Expression Assay in Breast Cancer. N. Engl. J. Med..

[48] Cardoso F., van’t Veer L.J., Bogaerts J., Slaets L., Viale G., Delaloge S., Pierga J.Y., Brain E., Causeret S., Delorenzi M. (2016). 70-Gene Signature as an Aid to Treatment Decisions in Early-Stage Breast Cancer. N. Engl. J. Med..

[49] Kalinsky K., Barlow W.E., Gralow J.R., Meric-Bernstam F., Albain K.S., Hayes D.F., Lin N.U., Hortobagyi G.N. (2021). 21-gene assay to inform chemotherapy benefit in node-positive breast cancer. N. Engl. J. Med..

[50] OPTIMA Optimal Personalised Treatment of Early Breast Cancer Using Multi-Parameter Analysis. http://optimabreaststudy.com/.

[51] Sparano J.A., Crager M.R., Tang G., Gray R.J., Stemmer S.M., Shak S. (2021). Development and Validation of a Tool Integrating the 21-Gene Recurrence Score and Clinical-Pathological Features to Individualize Prognosis and Prediction of Chemotherapy Benefit in Early Breast Cancer. J. Clin. Oncol..

[52] Sparano J.A., Crager M., Gray R.J., Tang G., Hoag J., Baehner F.L., Shak S., Makower D.F., Albain K.S., Hayes D.F. (2024). Clinical and Genomic Risk for Late Breast Cancer Recurrence and Survival. NEJM Evid..

[53] Foldi J., O’Meara T., Marczyk M., Sanft T., Silber A., Pusztai L. (2019). Defining Risk of Late Recurrence in Early-Stage Estrogen Receptor-Positive Breast Cancer. Clinical Versus Molecular Tools. J. Clin. Oncol..

[54] Early Breast Cancer Trialists’ Collaborative Group (EBCTCG) (2011). Relevance of breast cancer hormone receptors and other factors to the efficacy of adjuvant tamoxifen: Patient-level meta-analysis of randomised trials. Lancet.

[55] Cucciniello L., Gerratana L., Del Mastro L., Puglisi F. (2022). Tailoring adjuvant endocrine therapy in early breast cancer: When, how, and how long?. Cancer Treat. Rev..

[56] Gray R.G., Bradley R., Braybrooke J., Clarke M., Hills R.K., Peto R., Bergh J.C.S., Swain S.M., Davidson N.E., Francis P.A. (2023). Effects of ovarian ablation or suppression on breast cancer recurrence and survival: Patient-level meta-analysis of 14,993 pre menopausal women in 25 randomised trials. J. Clin. Oncol..

[57] Early Breast Cancer Trialists’ Collaborative Group (EBCTCG) (2015). Adjuvant bisphosphonate treatment in early breast cancer: Meta-analyses of individual patient data from randomised trials. Lancet.

[58] Tutt A.N., Garber J.E., Kaufman B., Viale G., Fumagalli D., Rastogi P., Gelber R.D., de Azambuja E., Fielding A., Balmaña J. (2021). Adjuvant Olaparib for Patients with *BRCA1*- or *BRCA2*-Mutated Breast Cancer. N. Engl. J. Med..

[59] A Study of Elacestrant Versus Standard Endocrine Therapy in Women and Men With ER+, HER2-, Early Breast Cancer With High Risk of Recurrence (ELEGANT). https://www.clinicaltrials.gov/study/NCT06492616.

[60] Riggio A.I., Varley K.E., Welm A.L. (2020). The lingering mysteries of metastatic recurrence in breast cancer. Br. J. Cancer.

[61] Davies C., Pan H., Godwin J., Gray R., Arriagada R., Raina V., Abraham M., Medeiros Alencar V.H., Badran A., Bonfill X. (2013). Long-term effects of continuing adjuvant tamoxifen to 10 years versus stopping at 5 years after diagnosis of oestrogen receptor-positive breast cancer: ATLAS, a randomised trial. Lancet.

[62] Zhang X.H., Giuliano M., Trivedi M.V., Schiff R., Osborne C.K. (2013). Metastasis dormancy in estrogen receptor-positive breast cancer. Clin. Cancer Res..

[63] Kim S., Tiedt R., Loo A., Horn T., Delach S., Kovats S., Haas K., Engstler B.S., Cao A., Pinzon-Ortiz M. (2018). The potent and selective cyclin-dependent kinases 4 and 6 inhibitor ribociclib (LEE011) is a versatile combination partner in preclinical cancer models. Oncotarget.

[64] Chen P., Lee N.V., Hu W., Xu M., Ferre R.A., Lam H., Bergqvist S., Solowiej J., Diehl W., He Y.-A. (2016). Spectrum and Degree of CDK Drug Interactions Predicts Clinical Performance. Mol. Cancer Ther..

[65] Rao P., Tullai J., Aspesi P., Mapa F., Cochran N., Sigoillot F., Roma G., Gleim S., Jacob J., Marchese J. Characterisation of Cancer Cell Lines Made Senescent by Exposure to Ribociclib, Doxorubicin, or TGFβ1, and Identification of Genes Required for Entry into Senescence and Senescent Cell Survival. Proceedings of the American Association for Cancer Research Annual Meeting 2021.

[66] Peuker C.A., Yaghobramzi S., Grunert C., Keilholz L., Gjerga E., Hennig S., Schaper S., Na I.-K., Keller U., Brucker S. (2021). Treatment with ribociclib shows favourable immunomodulatory effects in patients with hormone receptor-positive breast cancer—findings from the RIBECCA trial. Eur. J. Cancer.

[67] Hamilton E., Spring L., Fasching P. (2022). Pooled Analysis of Post-Progression Treatments after First-Line Endocrine Therapy in Patients with HR+/HER2- Advanced Breast Cancer in the MONALEESA-2, -3, and -7 Studies.

[68] Fleming G., Pagani O., Regan M., Walley B., Francis P. (2022). Adjuvant abemaciclib combined with endocrine therapy for high-risk early breast cancer: Updated efficacy and Ki-67 analysis from the monarchE study. Ann. Oncol..

[69] Johnston S.R.D., Toi M., O’Shaughnessy J., Rastogi P., Campone M., Neven P., Huang C.-S., Huober J., Jaliffe G.G., Cicin I. (2023). Abemaciclib plus endocrine therapy for hormone receptor-positive, HER2-negative, node-positive, high- risk early breast cancer (monarchE): Results from a preplanned interim analysis of a randomised, open-label, phase 3 trial. Lancet Oncol..

[70] Rastogi P., O’Shaughnessy J., Martin M., Boyle F., Cortes J., Rugo H.S., Goetz M.P., Hamilton E.P., Huang C.-S., Senkus E. (2024). Adjuvant Abemaciclib Plus Endocrine Therapy for Hormone Receptor–Positive, Human Epidermal Growth Factor Receptor 2–Negative, High-Risk Early Breast Cancer: Results From a Preplanned monarchE Overall Survival Interim Analysis, Including 5-Year Efficacy Outcomes. J. Clin. Oncol..

[71] Hortobagyi G.N., Lacko A., Sohn J., Cruz F., Borrego M.R., Manikhas A., Park Y.H., Stroyakocskiy D., Yardley D.A., Huang C.-S. (2024). A phase III trial of adjuvant ribociclib plus endocrine therapy vs endocrine therapy alone in patients with HR+/HER2- early breast cancer: Final invasive disease-free survival results from the NATALEE trial. Ann. Oncol..

[72] Fasching P.A. (2024). Adjuvant Ribociclib (RIB) Plus Nonsteroidal Aromatase Inhibitor (NSAI) in Patients (Pts) With HR+/HER2- Early Breast Cancer (EBC): 4-Year Outcomes From the NATALEE Trial. LBA13. Ann. Oncol..

[73] Slamon D.J., Fasching P.A., Hurvitz S., Chia S., Crown J., Martin M., Barrios C.H., Bardia A., Im S.A., Yardley D.A. (2023). Rationale and trial design of NATALEE: A Phase III trial of adjuvant ribociclib + endocrine therapy versus endocrine therapy alone in patients with HR+/HER2- early breast cancer. Ther. Adv. Med. Oncol..

[74] Schäffler H., Mergel F., Pfister K., Lukac S., Fink A., Veselinovic K., Rack B., Fink V., Leinert E., Dimpfl M. (2023). The Clinical Relevance of the NATALEE Study: Application of the NATALEE Criteria to a Real-World Cohort from Two Large German Breast Cancer Centers. Int. J. Mol. Sci..

[75] Kanjanapan Y., Anderson W., Smith M., Green J., Chalker E., Craft P. (2024). Real-World Analysis of Breast Cancer Patients Qualifying for Adjuvant CDK4/6 Inhibitors. Clin. Breast Cancer.

[76] Caswell-Jin J.L., Freedman R.A., Hassett M.J., Tang H., Garrett-Mayer E., Somefield M.R., Giordane S.H. (2024). Optimal Adjuvant Chemotherapy and Targeted Therapy for Early Breast Cancer-CDK4/6 Inhibitors: ASCO Rapid Guideline Update Clinical Insights. JCO Oncol. Pract..

[77] Nelson D.R., Brown J., Morikawa A., Method M. (2022). Breast cancer-specific mortality in early breast cancer as defined by high-risk clinical and pathologic characteristics. PLoS ONE.

[78] Lammers S.W.M., Meegdes M., Vriens I.J.H., Voogd A.C., Munck L.d., Nijnatten T.J.A.v., Keymeulen K.B.M.I., Tjan-Heijnen V.C.G., Geurts S.M.E. (2024). Treatment and survival of patients diagnosed with high-risk HR+/HER2- breast cancer in the Netherlands: A population-based retrospective cohort study. eSMO Open.

[79] Sheffield K.M., Peachey J.R., Method M., Grimes B.R., Brown J., Saverno K., Sugihara T., Cui Z.L., Lee K.T. (2022). A real-world US study of recurrence risks using combined clinicopathological features in HR-positive, HER2- negative early breast cancer. Future Oncol..

[80] Martín M., Carrasco E., Rodríguez-Lescure Á., Andrés R., Servitja S., Antón A., Ruiz-Borrego M., Bermejo B., Guerrero Á., Ramos M. (2023). Long-term outcomes of high-risk HR-positive and HER2-negative early breast cancer patients from GEICAM adjuvant studies and El Álamo IV registry. Breast Cancer Res. Treat..

[81] Tarantino P., Jin Q., Mittendorf E., King T., Curigliano G., Tolaney S.M. (2022). Clinical and pathological features of breast cancer patients eligible for adjuvant abemaciclib. Ann. Oncol..

[82] Fasching P.A., Hack C.C., Nabieva N., Maass N., Aktas B., Kümmel S., Thomssen C., Wolf C., Kolberg H.-C., Brucker C. (2024). Prognostic impact of selection criteria of current adjuvant endocrine therapy trials NATALEE and monarchE in postmenopausal HRpos/HER2neg breast cancer patients treated with upfront letrozole. Eur. J. Cancer.

[83] Giuliano A.E., Ballman K.V., McCall L., Beitsch P.D., Brennan M.B., Kelemen P.R., Ollila D.W., Hansen N.M., Whitworth P.W., Blumencranz P.W. (2017). Effect of Axillary Dissection vs. No Axillary Dissection on 10-Year Overall Survival Among Women With Invasive Breast Cancer and Sentinel Node Metastasis: The ACOSOG Z0011 (Alliance) Randomized Clinical Trial. JAMA.

[84] Donker M., van Tienhoven G., Straver M.E., Meijnen P., van de Velde C.J.H., E Mansel R., Cataliotti L., Westenberg A.H., Klinkenbijl J.H.G., Orzalesi L. (2014). Radiotherapy or surgery of the axilla after a positive sentinel node in breast cancer (EORTC 10981-22023 AMAROS): A randomised, multicentre, open-label, phase 3 non-inferiority trial. Lancet Oncol..

[85] Bartels S.A.L., Donker M., Poncet C., Sauvé N., Straver M.E., van de Velde C.J., Mansel R.E., Blanken C., Orzalesi L., Klinkenbijl J.H. (2023). Radiotherapy or Surgery of the Axilla After a Positive Sentinel Node in Breast Cancer: 10-Year Results of the Randomized Controlled EORTC 10981-22023 AMAROS Trial. J. Clin. Oncol..

[86] de Boniface J., Tvedskov T.F., Rydén L., Szulkin R., Reimer T., Kühn T., Kontos M., Gentilini O.D., Bagge R.O., Sund M. (2024). Omitting Axillary Dissection in Breast Cancer with Sentinel-Node Metastases. N. Engl. J. Med..

[87] Mittendorf E.A., King T.A., Tolaney S.M. (2022). Impact of RxPONDER and monarchE on the Surgical Management of the Axilla in Patients With Breast Cancer. J. Clin. Oncol..

[88] Williams A.D., Ruth K., Shaikh S.S., Vasigh M., Pronovost M.T., Aggon A.A., Porpiglia A.S., Bleicher R.J. (2024). Should patients with hormone receptor-positive, HER2-negative breast cancer and one or two positive sentinel nodes undergo axillary dissection to determine candidacy for adjuvant abemaciclib?. Cancer.

[89] Gaillard T., Piketty J., Feron J.G., Girard N., Pauly L., Gauroy E., Darrigues L., Grandal B., Pierga J.-Y., Hamy-Petit A.-S. (2024). Rethinking surgical revisions: Impact of the MonarchE trial on axillary dissection in hormone-positive HER2-negative early breast cancer patients potentially eligible for abemaciclib. Br. J. Cancer.

[90] Ahari D., Wilkinson M., Ali N., Taxiarchi V.P., Dave R.V., Gandhi A. (2024). Abemaciclib Therapy Using the MonarchE Criteria Results in Large Numbers of Excess Axillary Node Clearances-Time to Pause and Reflect?. Cancers.

[91] Rocco N., Ghilli M., Curcio A., Bortul M., Burlizzi S., Cabula C., Cabula R., Ferrari A., Folli S., Fortunato L. (2024). Is routine axillary lymph node dissection needed to tailor systemic treatments for breast cancer patients in the era of Is routine axillary lymph node dissection needed to tailor systemic treatments for breast cancer patients in the era of molecular oncology?. Eur. J. Surg. Oncol..

[92] de Boniface J., Appelgren M., Szulkin R., Alkner S., Andersson Y., Bergkvist L., Frisell J., Gentilini O.D., Kontos M., Kuhn T. (2024). Completion axillary lymph node dissection for the identification of pN2–3 status as an indication for adjuvant CDK4/6 inhibitor treatment: A post-hoc analysis of the randomised, phase 3 SENOMAC trial. Lancet Oncol..

[93] KISQALI® (Ribociclib) Prescribing Information. https://www.accessdata.fda.gov/drugsatfda_docs/label/2024/209092s018lbl.pdf.

[94] VERZENIO® (Abemaciclib) Prescribing Information. https://www.accessdata.fda.gov/drugsatfda_docs/label/2023/208716s010s011lbl.pdf.

[95] Fasching P.A., Slamon D., Nowecki Z., Kukielka-Budny B.D., Stroyakovsiy D., Yardley D., Huang C., Chan A., Chia S., Martin M. (2023). VP3–2023: Health-related quality of life (HRQoL) in the phase III NATALEE study of adjuvant ribociclib (RIB) plus a nonsteroidal aromatase inhibitor ( NSAI) vs NSAI alone in patients (pts) with HR+/HER2- early breast cancer (EBC). Ann. Oncol..

[96] Rugo H., O’shaughnessy J., Boyle F., Toi M., Broom R., Blancas I., Gumus M., Yamashita T., Im Y.-H., Rastogi P. (2022). Adjuvant abemaciclib combined with endocrine therapy for high-risk early breast cancer: Safety and patient-reported outcomes from the monarchE study. Ann. Oncol..

[97] Tolaney S.M., Guarneri V., Seo J.H., Curz J., Abreu M.H., Takahashi M., Barrios C., Mclntyre K., Wei R., Munoz M. (2024). Long-term patient-reported outcomes from monarchE: Abemaciclib plus endocrine therapy as adjuvant therapy for HR +, HER2-, node-positive, high-risk, early breast cancer. Eur. J. Cancer.

[98] Mayer E., Smith M.L., Guerin A., Latremouille-Viau D., Hazra N.C., Meng Y., Qu W., Bellefleur R., Ganapathy V., Santarsiero L. (2024). Patient preferences for CDK4/6 inhibitor treatments in HR+/HER2- early breast cancer: A discrete choice survey study. Cancer Res..

[99] FDA Approves Abemaciclib with Endocrine Therapy for Early Breast Cancer. https://www.fda.gov/drugs/resources-information-approved-drugs/fda-approves-abemaciclib-endocrine-therapy-early-breast-cancer.

[100] Royce M., Osgood C., Mulkey F., Bloomquist E., Pierce W.F., Roy A., Kalavar S., Ghosh S., Philip R., Rizvi F. (2022). FDA Approval Summary: Abemaciclib With Endocrine Therapy for High-Risk Early Breast Cancer. J. Clin. Oncol..

[101] FDA Expands Early Breast Cancer Indication for Abemaciclib with Endocrine Therapy. https://www.fda.gov/drugs/resources-information-approved-drugs/fda-expands-early-breast-cancer-indication-abemaciclib-endocrine-therapy.

[102] Meirson T., A Goldstein D., Gyawali B., Tannock I.F. (2023). Review of the monarchE trial suggests no evidence to support use of adjuvant abemaciclib in women with breast cancer. Lancet Oncol..

[103] Loi S., Curigliano G., Salgado R.F., Diaz R.I.R., Delaloge S., Rojas C., Kok M., Manich C.S., Harbeck N., Mittendorf E.A. (2023). LBA20 A randomized, double-blind trial of nivolumab (NIVO) vs placebo (PBO) with neoadjuvant chemotherapy (NACT) followed by adjuvant endocrine therapy (ET) ± NIVO in patients (pts) with high-risk, ER+ HER2- primary breast cancer (BC). Ann. Oncol..

[104] Cardoso F., McArthur H.L., Schmid P., Cortes J., Harbeck N., Telli M.L., Cescon D.W., O’Shaughnessy J., Fasching P., Shao Z. (2023). LBA21 KEYNOTE-756: Phase III study of neoadjuvant pembrolizumab (pembro) or placebo (pbo) + chemotherapy (chemo), followed by adjuvant pembro or pbo + endocrine therapy (ET) for early-stage high-risk ER+/HER2- breast cancer. Ann. Oncol..

[105] Masuda J., Sakai H., Tsurutani J., Tanabe Y., Masuda N., Iwasa T., Takahashi M., Futamura M., Matsumoto K., Aogi K. (2023). Efficacy, safety, and biomarker analysis of nivolumab in combination with abemaciclib plus endocrine therapy in patients with HR-positive HER2-negative metastatic breast cancer: A phase II study (WJOG11418B NEWFLAME trial). J. Immunother. Cancer.

[106] Rugo H.S., Kabos P., Beck J.T., Jerusalem G., Wildiers H., Sevillano E., Paz-Ares L., Chisamore M.J., Chapman S.C., Hossain A.M. (2022). Abemaciclib in combination with pembrolizumab for HR+, HER2− metastatic breast cancer: Phase 1b study. npj Breast Cancer.

[107] Jerusalem G., Prat A., Salgado R., Reinisch M., Saura C., Ruiz-Borrego M., Nikolinakos P., Ades F., Filian J., Huang N. (2023). Neoadjuvant nivolumab + palbociclib + anastrozole for oestrogen receptor-positive/human epidermal growth factor receptor 2-negative primary breast cancer: Results from CheckMate 7A8. Breast.

[108] Turner N.C., Marme F., Kim S.-B., Bonnefoi H., Garcia-Saenz J.A., Torres A.A., Bear H., Tesch H., Olive M.M., McCarthy N. Detection of circulating tumour DNA following neoadjuvant chemotherapy and surgery to anticipate early relapse in ER positive and HER2 negative breast cancer: Analysis from the PENELOPE-B trial. Proceedings of the ASCO 2023 Annual Meeting.

[109] Loi S., Johnston S.R.D., Arteaga C.L., Graff S.L., Chandarlapaty S., Goetz M.P., Desmedt C., Sasano H., Liu D., Rodrik-Outmezguine V. (2024). Prognostic utility of ctDNA detection in the monarchE trial of adjuvant abemaciclib plus endocrine therapy (ET) in HR+, HER2-, node-positive, high-risk early breast cancer (EBC). J. Clin. Oncol..

